# Novel Diamond Films Synthesis Strategy: Methanol and Argon Atmosphere by Microwave Plasma CVD Method Without Hydrogen

**DOI:** 10.1186/s11671-016-1628-x

**Published:** 2016-09-20

**Authors:** Li Yang, Caiyi Jiang, Shenghui Guo, Libo Zhang, Jiyun Gao, Jinhui Peng, Tu Hu, Liang Wang

**Affiliations:** 1 State International Joint Research Center of Advanced Technology for Superhard Materials, Kunming University of Science and Technology, Kunming, 650093 China; 2 State Key Laboratory of Complex Nonferrous Metal Resources Clean Utilization, Kunming University of Science and Technology, Kunming, 650093 China; 3 National Local Joint Laboratory of Engineering Application of Microwave Energy and Equipment Technology, Kunming, Yunnan 650093 China; 4 School of Chemistry and Environment, Yunnan Minzu University, Kunming, 650500 China; 5 Faculty of Metallurgical and Energy Engineering, Kunming University of Science and Technology, Kunming, Yunnan 650093 China

**Keywords:** Diamond films, Microwave plasma CVD, Methanol, Synthesis strategy

## Abstract

Diamond thin films are grown on silicon substrates by only using methanol and argon mixtures in microwave plasma chemical vapor deposition (MPCVD) reactor. It is worth mentioning that the novel strategy makes the synthesis reaction works smoothly without hydrogen atmosphere, and the substrates temperature is only 500 °C. The evidence of surface morphology and thickness under different time is obtained by characterizing the samples using scanning electron microscopy (SEM). X-ray diffractometer (XRD) spectrum reveals that the preferential orientation of (111) plane sample is obtained. The Raman spectra indicate that the dominant component of all the samples is a diamond. Moreover, the diamond phase content of the targeted films was quantitatively analyzed by X-ray photoelectron spectroscopy (XPS) method, and the surface roughness of diamond films was investigated by atomic force microscope (AFM). Meanwhile, the possible synthesis mechanism of the diamond films in methanol- and argon-mixed atmosphere was discussed.

## Background

Diamond films have been widely used in optical, mechanical, electrical, and biology fields because of its excellent physical and chemical properties [1, 2]. For instance, the extreme hardness and low friction coefficient provide the flexibility of the material for protective coatings [3]; the remarkable hardness and high optical transparency in a broad wavelength range provide an ideal material for infrared windows [4]; and the intrinsic electrical insulation or superconductivity after *p*-doping makes these attractive for electronic material because of its high thermal conductivity [5].

Nowadays, the main methods of diamond films preparation are chemical vapor deposition (CVD) and physical vapor deposition [6]. Meanwhile, CVD method shows many advantageous features including simple film-forming equipment and high purity and uniformity of the diamond films. In general, CVD method is composed of hot filament CVD, oxyacetylene combustion flame, DC plasma jet CVD, and microwave plasma CVD [7]. The technique of microwave plasma CVD is most widely used to synthesize diamond films with high purity and uniformity [8]. However, these synthesis methods of diamond films frequently rely on the high-pressure and high-purity hydrogen or methane, which have the potential risk and are high cost. For example, the ultrananocrystalline diamond film was synthesized by Ar–H_2_–CH_4_ gas mixture [9]; the nanocrystalline diamond film was prepared by Ar–H_2_–CH_4_–O_2_ gas mixture [10]; and the microcrystalline diamond was grown in H_2_–CH_4_ gas mixture [11]. In order to reduce the usage of hazardous gas, the investigator tries to use the liquid diffusion as a carbon source for synthetic diamond films. For example, the diamond films were synthesized by using ethanol and acetone as the carbon source [12, 13]. But it is obvious that the high-purity H_2_ is a necessary feed gas. Thus, it is a valuable exploration on how to avoid the risk gas for the synthesis of diamond films.

To obtain diamond films under the condition of no risk gas participation, we conducted a novel strategy. We adopt the method of liquid diffusion and methanol as a carbon source for diamond films synthesis. The most advantage is that under the atmosphere of argon, CH_3_OH was dissociated into C_2_, CH_3_, CH, H, and OH radicals in the plasma under the condition of microwave energy [14], and it is beneficial to promote the synthesis of the diamond films. Thus, the novel method can synthesize diamond films away from the dependence on risk gas drastically. In this work, the diamond films were synthesized by methanol (CH_3_OH) and argon (Ar) mixtures using microwave plasma CVD. The morphology, the structure, the thickness, the surface roughness, and the diamond phase content of the films were analyzed by scanning electron microscopy (SEM), X-ray diffractometer (XRD), atomic force microscope (AFM), and Raman spectroscopy, respectively. Meanwhile, the content of diamond phase with sp^3^ hybridized orbital was evaluated by X-ray photoelectron spectroscopy (XPS), and the diamond growth mechanism under CH_3_OH-Ar atmosphere was discussed.

## Methods

### Seeding and Experiment Design

All chemicals used were of analytical grade without further purification. In order to improve nucleation density, polished commercial *p*-type (100) silicon wafer (2 in. in diameter, 200 ± 10 μm in thickness) was scratched with 0.25 μm diamond powders for 45 min by ultrasonic. Then, the silicon wafer was cleaned in acetone, alcohol, and deionizer water for 10 min by ultrasonic. Finally, the nitrogen gas was used to dry the silicon wafer.

Diamond films were synthesized in a 2.45-GHz, 8-kW MPCVD system [1], and the liquid CH_3_OH was introduced into reaction chamber by Ar bubbling. All experiments were enforced with the following steps strictly. First, the background vacuum of the reaction chamber was realized by the extraction of the superfluous gases until the pressure reduced to 0.1 Pa and then high-purity Ar gas (99.999 %) with 200 sccm was introduced. Then, the silicon substrate was treated for 20 min in Ar plasma under the condition of the microwave output power of 1000 W and the reaction chamber press of 2 kPa [15]. Finally, the gaseous CH_3_OH was introduced by 5 sccm argon gas flows through the pure liquid CH_3_OH, in the meanwhile, the previously set Ar gas flow kept constant (200 sccm). The diamond film was deposited under the fixed conditions of reaction chamber press (15 kPa), microwave power (1.8 kW), and substrate temperature (~500 °C) for 2, 4, 6, 12 , and 24 h.

### Characterization

The microstructures, crystal structure, and the thickness of the diamond films were investigated by XRD (Bruker D8 X-ray diffractometer with Cu-Kα radiation source, *λ* = 1.5406 Å), SEM (JSM-6701F with an accelerating voltage of 15 kV, JEOL), respectively. The surface roughness of diamond films was investigated by AFM (Bruker Dimension Icon), and Raman spectra (Raman spectrometer, LabRAM HR Evolution with ~1-μm spot size using a 532-nm diode laser source) was adopted to explore the film quality of diamond samples and the relative content of diamond phase (sp^3^ type hybridized space structure) was further analyzed by XPS (AXIS-ULTRA DLD-600 W photoelectron spectrometer with Al *K*1 radiation).

## Results and Discussion

### Surface Morphology and Structure

**Fig. 1:**
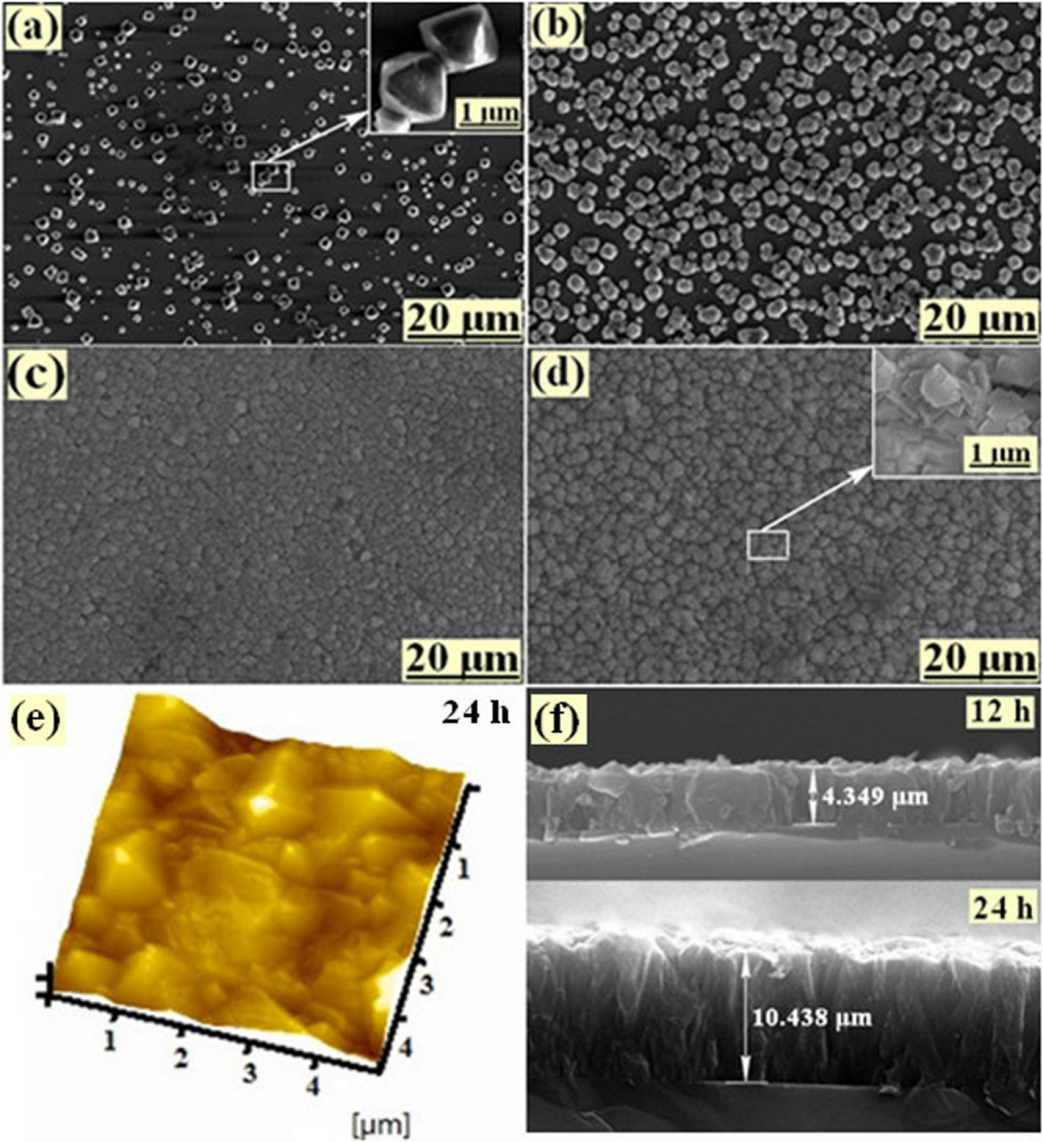
Morphology image of diamond films. SEM of surface: **a** 2, **b** 4, **c** 6, and **d** 12 h; SEM of cross section: **f** 12 and 24 h; AFM: **e** 24 h

For a morphology overview, Fig. 1a–d shows the SEM images of the diamond surface morphology, which demonstrated a significant change while in the increase of the deposition time from 2 to 12 h. Figure 1a shows the typical morphology of diamond grown for 2 h on *p*-type (100) silicon wafer, and the nucleation density is approximately 1.5 × 10^8^ cm^−2^. In particular, the diamond particles are independent of each other with many voids and low uniformity. The illustration from Fig. 1a clearly shows the top morphology of diamond particles, which appears (400) and (111) oriented lattice plane [16, 17]. To all appearances, the flank of triangular and planar top of diamond particle were (111) and (400) oriented lattice plane, respectively. In further observation, from Fig. 1a–d), it is observed that the voids are reduced gradually with the increase of deposition time. And the secondary nucleation rate of diamond films increases when the deposition time reaches 4 h, as shown in Fig. 1b. The diamond particles fully cover the silicon wafer when the diamond was grown for 12 h. Moreover, the edges and corners of diamond particles disappear with the grown time. It indicates that the growth rate of the diamond is restricted by the secondary nucleation rate [18]. The thickness of diamond films was tested by SEM of cross section, as shown in Fig. 1f. From this picture, the thickness of diamond films is 4.349 and 10.438 μm under the growth condition of 12 and 24 h, respectively. Thus, the difference of thickness is 6.089 μm, and the growth rate is approximately 0.507 μm/h under the CH_3_OH-Ar-mixed atmosphere. In addition, the surface roughness is an important parameter for diamond films. In our work, the surface roughness was investigated by AFM, as shown in Fig. 1e. From this picture, the surface quality of diamond films for 24-h growth is good and the surface roughness is only 89.87 nm.

**Fig. 2:**
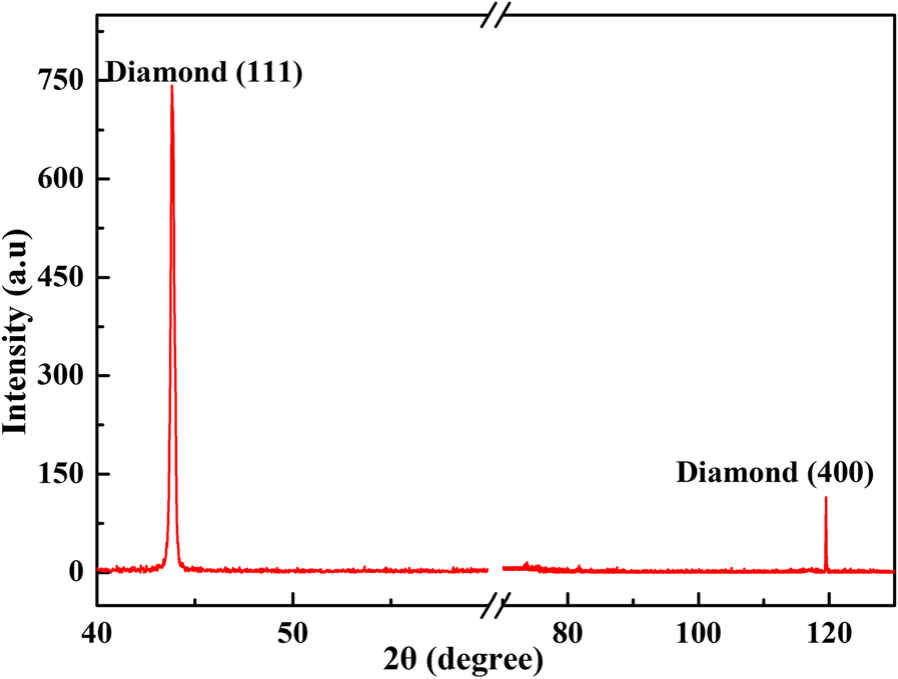
X-ray diffraction spectrum of diamond grown for 12 h

The crystal structure of diamond film grown for 12 h was investigated using XRD method, as shown in Fig. 2. In order to better analyze the diffraction information of diamond film, the characteristic peak of silicon was ignored from 67° to 72°. From Fig. 2, there are two primary peaks at the diffraction angle 2*θ* of 43.94° and 119.58°, which corresponds to (111) and (400) reflections of the diamond [15], respectively. It indicates that the diamond shown polycrystalline nature and preferential (111) texture consistent with the result of SEM analysis. The average grain size is 42.6 nm, which can be calculated using the Sherrer equation [19]: *D =* 0.89*λ/B*cos*θ*. Where *λ* =0.154 nm, *B* is the full width at half maximum of (111) diffraction peak.

### Quality Evaluation of Diamond Films

**Fig. 3:**
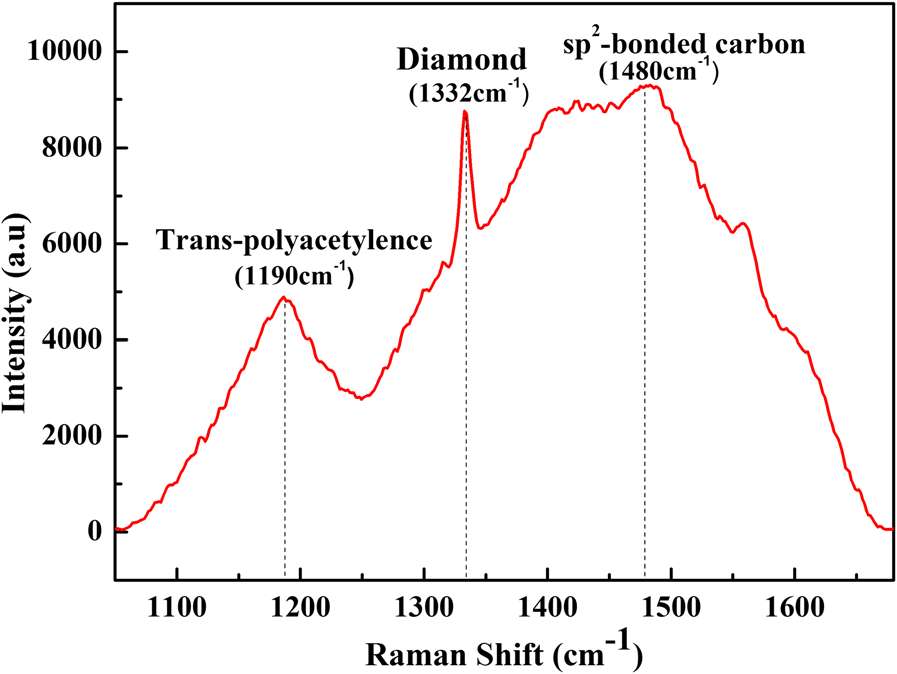
Raman spectra of diamond grown for 12 h

In order to confirm qualitatively the bonding information of carbon atoms, Raman spectrum method was adopted. Raman is a scattering spectrum from the inelastic collision between photon and molecule, and closely related with bond length and bond angle of the atom. Moreover, different substance has its own intrinsic Raman shift, which can make qualitative analysis for material. In particular, Raman detection is sensitive to carbon material, and the sensitivity for sp^2^ hybridization components is 50 times of sp^3^ hybridization components [20]. Thus, the bonding information of diamond film is widely evaluated by Raman spectra. Figure 3 shows Raman spectra of the diamond films grown for 12 h. From this picture, there are three primary peaks at 1190, 1332, 1480 cm^−1^ around, which attribute to trans-polyacetylene (t-PA), diamond, and sp^2^-bonded carbon [21, 22], respectively. The sharp peak with small FWHM has 1332 cm^−1^ around and has no significant graphite peak (around 1580 cm^−1^), indicating that the crystalline of diamond is excellent [23].

**Fig. 4:**
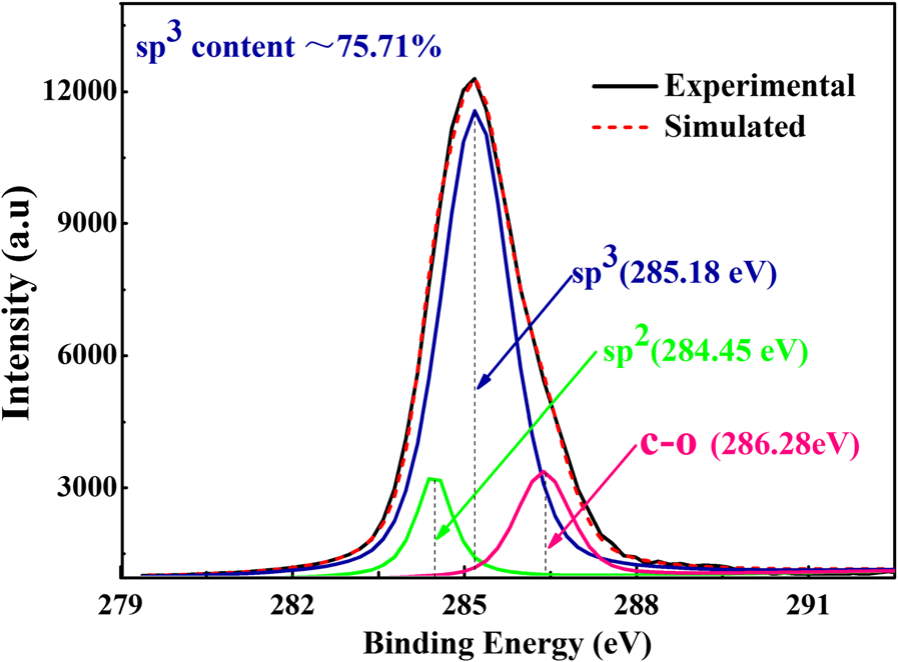
High-resolution XPS spectra of diamond grown for 12 h

On the basis of Raman analysis results, XPS test was adopted to further uncover the quantitative information about the relative content of diamond phase because it can distinguish between sp^2^ and sp^3^ hybridized space structure of carbon atoms in the material [24]. Thus, XPS is used to support the result of Raman spectrum from the electronic level. Figure 4 shows the high-resolution XPS C1s spectra of diamond, and the spectrum is divided into three components according to the evidence of Raman test. The three peaks of C1s were 285.18, 284.45, and 286.28 eV, which correspond to sp^3^, sp^2^, and C–O chemical bonds [25, 26], respectively. Meanwhile, the calculated relative content of sp^3^ (diamond phase) is approximately 75.71 % by Multipak V9.3 software, which is well consistent with Raman analysis.

### Possible Synthesis Mechanism of Diamond Films

**Fig. 5:**
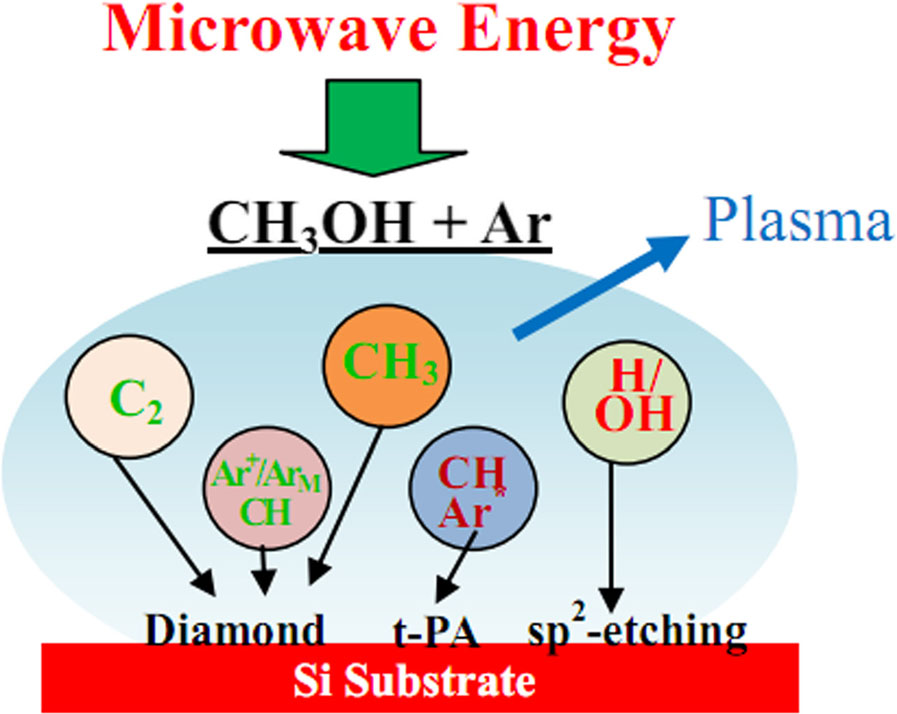
Possible synthesis mechanism of diamond films

Equations (1)–(10) proposes a possible mechanism of diamond films synthesis by using CH_3_OH-Ar-mixed atmosphere in microwave plasma CVD reactor. On the one hand, gaseous Ar was converted to Ar*, Ar+, Ar_M_ (metastable argon) by means of the stimulation of microwave energy, as shown in Eqs.(1)–(3). On the other hand, CH_3_OH gas was dissociated into C_2_, CH_3_, CH, H, and OH radicals followed by Eq. (4) in the plasma under the condition of microwave energy [27, 28]. It is worth mentioning that –CH_3_ species has sp^3^ steric configuration, which is similar to the lattice orientation of diamond. It provides the possibility to facilitate the critical architecture of carbon skeletons. The –CH_3_ species reach the surface of substrate with an open site or dangling bond by convection and diffusion movement [29]. Under the excitation of microwave energy, carbon-hydrogen bonds are dissociated to reconfigure the diamond or non-diamond carbon phase after a series of complex reaction [30]. Meanwhile, the non-diamond carbon phase, such as graphite and diamond-like carbon, were removed by H, OH radicals followed by Eq. (10) [31, 7]. According to Eq. (5), CH radicals reciprocally combined into C_2_H_2_ [32]. Afterwards, as indicated in Eqs.(6)–(9), there are three evolutionary paths of C_2_H_2_ via the reaction with Ar^*^ radical, Ar^+^, and Ar_M_ to create C_2_H^+^ and highly active C_2._ The radical C_2_ prefers to graft in sp^3^ carbon skeletons in the low binding energy barrier (~6 kcal/mol), which indicate the process does not need open site or dangling bond from H atom bombardment [33]. Thus, the diamond film can grow smoothly even under the environment lack of H_2_. The possible mechanism for diamond film synthesis is shown in Fig. 5.

1 $$ \mathrm{A}\mathrm{r} + \mathrm{e}\ \to \mathrm{A}{\mathrm{r}}_{\mathrm{M}}+\mathrm{e} $$

2 $$ \mathrm{A}\mathrm{r} + \mathrm{e}\ \to \mathrm{A}\mathrm{r}* + \mathrm{e} $$

3 $$ \mathrm{A}\mathrm{r} + \mathrm{e}\ \to \mathrm{A}{\mathrm{r}}^{+}+\mathrm{e} $$

4 $$ \mathrm{C}{\mathrm{H}}_3\mathrm{O}\mathrm{H}\ \to\ {\mathrm{C}}_2 + \mathrm{C}{\mathrm{H}}_3 + \mathrm{C}\mathrm{H} + \mathrm{H} + \mathrm{O}\mathrm{H} $$

5 $$ \mathrm{C}\mathrm{H} + \mathrm{C}\mathrm{H}\ \to\ {\mathrm{C}}_2{\mathrm{H}}_2 $$

6 $$ {\mathrm{C}}_2{\mathrm{H}}_2 + \mathrm{A}\mathrm{r}*\ \to\ {\mathrm{C}}_2{{\mathrm{H}}_2}^{+} + \mathrm{A}\mathrm{r} $$

7 $$ {\mathrm{C}}_2{\mathrm{H}}_2 + \mathrm{A}{\mathrm{r}}_{\mathrm{M}}\to\ {\mathrm{C}}_2 + {\mathrm{H}}_2 + \mathrm{A}\mathrm{r} $$

8 $$ {\mathrm{C}}_2{\mathrm{H}}_2 + \mathrm{A}{\mathrm{r}}^{+}\to\ {\mathrm{C}}_2{\mathrm{H}}^{+} + \mathrm{A}\mathrm{r} $$

9 $$ {\mathrm{C}}_2{\mathrm{H}}^{+} + \mathrm{e}\ \to\ {\mathrm{C}}_2+\mathrm{H} $$

10 $$ {\mathrm{C}}_{\mathrm{g}}+\mathrm{H}\to {\mathrm{C}}_{\mathrm{d}} + {\mathrm{H}}_2 $$

## Conclusions

A novel strategy was proposed to synthesize diamond films by only using methanol and argon mixtures by MPCVD method under the deposition temperature of 500 °C. The new fabrication method escaped from dependence on the inflammable and explosive high-express hydrogen or methane. The analytical results indicate that the diamond films have a good crystalline quality with preferential (111) lattice plane, and the diamond phase content is approximately 75.71 %. The possible mechanism reveals that CH_3_OH gas was dissociated into CH_3_ with sp^3^ steric configuration, which is important for diamond phase synthesis. Meanwhile, CH_3_OH gas was dissociated also into H and OH radicals, which can improve the quality of the diamond.
